# Blood transcriptomics to facilitate diagnosis and stratification in pediatric rheumatic diseases – a proof of concept study

**DOI:** 10.1186/s12969-022-00747-x

**Published:** 2022-10-17

**Authors:** My Kieu Ha, Esther Bartholomeus, Luc Van Os, Julie Dandelooy, Julie Leysen, Olivier Aerts, Vasiliki Siozopoulou, Eline De Smet, Jan Gielen, Khadija Guerti, Michel De Maeseneer, Nele Herregods, Bouchra Lechkar, Ruth Wittoek, Elke Geens, Laura Claes, Mahmoud Zaqout, Wendy Dewals, Annelies Lemay, David Tuerlinckx, David Weynants, Koen Vanlede, Gerlant van Berlaer, Marc Raes, Helene Verhelst, Tine Boiy, Pierre Van Damme, Anna C. Jansen, Marije Meuwissen, Vito Sabato, Guy Van Camp, Arvid Suls, Jutte Van der Werff ten Bosch, Joke Dehoorne, Rik Joos, Kris Laukens, Pieter Meysman, Benson Ogunjimi

**Affiliations:** 1 Center for Health Economics Research and Modelling Infectious Diseases (CHERMID), Vaccine and Infectious Disease Institute, University of Antwerp, Wilrijk, Belgium; 2 Antwerp Unit for Data Analysis and Computation in Immunology and Sequencing (AUDACIS), University of Antwerp, Antwerp, Belgium; 3 Antwerp Center for Translational Immunology and Virology (ACTIV), Vaccine and Infectious Disease Institute, University of Antwerp, Wilrijk, Belgium; 4 Center of Medical Genetics, University of Antwerp, Antwerp University Hospital, Edegem, Belgium; 5 Ophthalmology Department, Antwerp University Hospital, Edegem, Belgium; 6 Dermatology Department, Antwerp University Hospital, Edegem, Belgium; 7 Department of Translational Research in Immunology and Inflammation, University of Antwerp, Wilrijk, Belgium; 8 Pathology Department, Antwerp University Hospital, Edegem, Belgium; 9 Radiology Department, Antwerp University Hospital, Edegem, Belgium; 10 Department of Molecular – Morphology – Microscopy, University of Antwerp, Wilrijk, Belgium; 11 Clinical Biology Department, Antwerp University Hospital, Edegem, Belgium; 12 Radiology Department, Brussels University Hospital, Jette, Belgium; 13 Radiology Department, Ghent University Hospital, Ghent, Belgium; 14 Department of Immunology, Allergology, and Rheumatology, Antwerp University Hospital, Edegem, Belgium; 15 Rheumatology Department, Ghent University Hospital, Ghent, Belgium; 16 Rheumatology Department, Antwerp Hospital Network, Antwerp, Belgium; 17 Pediatric Neurology Unit, Antwerp University Hospital, Edegem, Belgium; 18 Pediatric Cardiology Department, Antwerp University Hospital, Edegem, Belgium; 19 Pediatric Cardiology Department, Antwerp Hospital Network, Antwerp, Belgium; 20 Department of Pediatrics, Turnhout General Hospital, Turnhout, Belgium; 21 Department of Pediatrics, Catholic University of Louvain, Louvain-la-Neuve, Belgium; 22 Department of Pediatrics, Namur University Hospital Center, Site Dinant, Dinant, Belgium; 23 Department of Pediatrics, Namur University Hospital Center, Site Sainte-Elisabeth, Namur, Belgium; 24 Department of Pediatrics, Nikolaas General Hospital, Sint-Niklaas, Belgium; 25 Department of Emergency Medicine/Pediatric Care, Brussels University Hospital, Jette, Belgium; 26 Department of Pediatrics, Jessa Hospital, Hasselt, Belgium; 27 Department of Pediatric Neurology, Ghent University Hospital, Ghent, Belgium; 28 Department of Pediatric Rheumatology, Antwerp University Hospital, Edegem, Belgium; 29 Center for the Evaluation of Vaccine, Vaccine and Infectious Disease Institute, University of Antwerp, Wilrijk, Belgium; 30 Antwerp Center for Pediatric Rheumatology and Autoinflammatory Diseases, Antwerp, Belgium; 31 Pediatric Immunology Department, Brussels University Hospital, Jette, Belgium; 32 Department of Pediatric Rheumatology, Ghent University Hospital, Ghent, Belgium; 33 ADREM Data Lab, Department of Mathematics and Computer Science, University of Antwerp, Antwerp, Belgium; 34 Biomedical Informatics Research Network Antwerp, University of Antwerp, Antwerp, Belgium; 35 Department of Pediatric Rheumatology, Brussels University Hospital, Jette, Belgium

**Keywords:** Pediatric rheumatic diseases, RNA sequencing, Blood transcriptomics, Classification model

## Abstract

**Background:**

Transcriptome profiling of blood cells is an efficient tool to study the gene expression signatures of rheumatic diseases. This study aims to improve the early diagnosis of pediatric rheumatic diseases by investigating patients’ blood gene expression and applying machine learning on the transcriptome data to develop predictive models.

**Methods:**

RNA sequencing was performed on whole blood collected from children with rheumatic diseases. Random Forest classification models were developed based on the transcriptome data of 48 rheumatic patients, 46 children with viral infection, and 35 controls to classify different disease groups. The performance of these classifiers was evaluated by leave-one-out cross-validation. Analyses of differentially expressed genes (DEG), gene ontology (GO), and interferon-stimulated gene (ISG) score were also conducted.

**Results:**

Our first classifier could differentiate pediatric rheumatic patients from controls and infection cases with high area-under-the-curve (AUC) values (AUC = 0.8 ± 0.1 and 0.7 ± 0.1, respectively). Three other classifiers could distinguish chronic recurrent multifocal osteomyelitis (CRMO), juvenile idiopathic arthritis (JIA), and interferonopathies (IFN) from control and infection cases with AUC ≥ 0.8. DEG and GO analyses reveal that the pathophysiology of CRMO, IFN, and JIA involves innate immune responses including myeloid leukocyte and granulocyte activation, neutrophil activation and degranulation. IFN is specifically mediated by antibacterial and antifungal defense responses, CRMO by cellular response to cytokine, and JIA by cellular response to chemical stimulus. IFN patients particularly had the highest mean ISG score among all disease groups.

**Conclusion:**

Our data show that blood transcriptomics combined with machine learning is a promising diagnostic tool for pediatric rheumatic diseases and may assist physicians in making data-driven and patient-specific decisions in clinical practice.

**Supplementary Information:**

The online version contains supplementary material available at 10.1186/s12969-022-00747-x.

## Background

Pediatric rheumatic diseases encompass a spectrum of autoimmune and autoinflammatory diseases that can affect the joints, muscles, bones, and other organs in children under the age of 16–18 years. Although many pediatric rheumatic diseases typically present with joint manifestations, other organs, including the eyes, skin, muscles, and gastrointestinal tract, may also be affected. Children who present with rheumatic symptoms often pose several challenges to their physicians. First, typical symptoms such as fever, rash, redness, pain and/or swelling at joints are common in many rheumatic diseases: (i) a transient/self-limiting process such as (reactive) infectious arthritis, (ii) a relapsing process like auto-inflammatory diseases (AID), (iii) a chronic condition like vasculitis, chronic recurrent multifocal osteomyelitis (CRMO), or juvenile idiopathic arthritis (JIA), (iv) an interferonopathy (IFN) such as dermatomyositis or systemic lupus erythematosus (SLE) characterized by dysregulation of type I interferon, or (v) diseases related to the human leukocyte antigen B51 serotype (HLA-B51). Second, once a disease has been confirmed, it is still difficult to make a rapid and definitive classification amongst the different subtypes within the disease due to its rarity and clinical presentation variability. This has challenged physicians’ efforts in making specific diagnoses and assigning proper treatments.

Transcriptome profiling of blood cells has proven to be useful and efficacious in identifying gene expression signatures in rheumatic diseases [1, 2], enabling physicians to make data-driven and patient-specific decisions. Thanks to the use of transcriptomics, the participation of type I interferons participate in the pathophysiology of SLE [3] and dermatomyositis [4] was discovered and has become one of the paramount findings in rheumatology. Psoriatic arthritis is another exemplary disease demonstrating the usefulness of applying transcriptomics to identify important pathways related to interleukins IL-12, IL-17 and IL-23 [1]. Large datasets of gene expression generated from such studies are highly interesting, yet their systematic analysis and interpretation is quite challenging. Thus, there has been considerable interest in applying machine learning on blood cells’ gene expression in order to obtain new insights into the pathophysiology of rheumatic diseases which in turn may have important implications for their clinical management [5, 6].

By investigating the whole blood gene expression of children with rheumatic diseases in comparison with reactive/post-infection controls, we aim to develop computational classifiers based on the obtained transcriptome data that allow us to identify pediatric patients with rheumatic diseases and distinguish different rheumatic groups (e.g., CRMO, JIA, and IFN), and thus, ultimately, to improve the diagnosis of future patients.

## Methods

### Patients and controls

After obtaining written consent, 48 children (1 to 16 years old) with rheumatic diseases (i.e., AID, CRMO, IFN, JIA, vasculitis, and HLA-B51 related rheumatic diseases) were recruited, prior to any treatment except non-steroidal anti-inflammatory drugs (NSAIDs), from May 2016 until August 2020, at the Divisions of Pediatric Rheumatology of four hospitals in Belgium (Antwerp Hospital Network, Antwerp University Hospital, Brussels University Hospital, and Ghent University Hospital). Venous blood was collected into PAXgene® Blood RNA tubes (PreAnalytiX, Switzerland). Clinical details of the patients can be found in Table S1. As controls, 46 children with PCR-confirmed viral (mainly enterovirus) infections were also recruited and requested to provide blood twice: first while being actively infected, and second following remission [7]. Only 35 children agreed to a second venapunction as it was not obligatory.

### RNA extraction

PAXgene® Blood RNA tubes were kept at -80 °C within 72 h after blood collection until use. RNA extraction was performed via a column-based RNA extraction using the PAXgene® Blood RNA extraction kit (Qiagen, Germany). To optimize RNA concentrations, we used the RNA Clean & Concentrator™-5 kit (Zymo Research, USA). We verified the RNA quality using the RNA ScreenTape Analysis on the Tapestation (Agilent, USA).

### 3’ mRNA library preparation and sequencing

All RNA samples were prepared with the QuantSeq3′ mRNA-Seq Library Prep Kit for Illumina (Lexogen GmbH, Austria) following the standard supplier’s protocol for long fragments. During the RNA removal step, we also added globin blockers, so none of the abundant globin mRNA was copied to double stranded cDNA. The resulting amplified cDNA libraries were equimolarly pooled and sequenced on NextSeq 500 (high output v2,5 kit, 150 cycli, single read) (Illumina, USA) with up to 40 samples per batch. This gave us an optimum of 10 million reads for each sample. All samples were prepared and sequenced in 4 batches.

### Raw data processing

Raw data from the NextSeq was demultiplexed and further processed per batch through an in-house pipeline. The quality of all reads was evaluated using FastQC (v0.11.9) before and after processing with Trimmomatic (v0.36). Trimmomatic removed the leading 20 bases from reads, ensure a minimum quality score of 15 over a sliding window of 4 bases and required a minimum read length of 30 bases. As usage of oligodT primers might cause poly-A stretches at the 3′ end, the latter end was trimmed with our own in-house poly-A removal script. All sequences that remained after trimming were mapped against the human reference genome build 38 (polymorph variants excluded) with HISAT2 (v2.0.4). HTseq (v0.6.1) was used to count all reads for each gene and generate a readcount table.

### Cluster identification

Clustering is an unsupervised learning algorithm that is used to discover patterns in high-dimensional data that would not be easily identified by conventional statistics. The readcount table obtained after raw data processing was normalized by median scaling. Then, the t-distributed stochastic neighbor embedding (t-SNE) method and hierarchical clustering algorithms were applied on the normalized readcount data to assign data into different clusters based on the (dis)similarity in the gene expression between samples.

### Differential gene expression and gene ontology enrichment analyses

Preliminary filtering of the normalized readcount data was performed by removing genes with fewer than 10 readcounts over all samples. Differential gene expression analyses were performed using the DESeq2 Bioconductor package in the open-source statistical software R. To account for batch effects, the batch number was included in the DESeq2 design. Differentially expressed genes with log2 fold change ≥ 2 (either up- or down-regulated) and p-value < 0.01 (adjusted for multiple testing by the False Discovery Rate method) were passed on to gene ontology enrichment analysis using the topGO package in R. Fisher’s exact test was performed to determine significantly enriched/depleted gene ontology terms relating to biological processes.

### Classifier development

Due to the larger number of genes in the dataset, a feature selection step was performed using the Boruta package in R [8] with a Bonferroni correction to identify genes that had good predictive power for disease classification. The Boruta algorithm was applied on the normalized gene expression values obtained from DESeq2. With the Boruta selected genes, classification models were trained using the Random Forest algorithm [9], also in R. Validation of the trained classifiers was performed using a leave-one-out cross-validation strategy. In this strategy, a single sample would be removed from the dataset and a classification model was trained on the remaining samples. The model was then used to classify the left-out sample. This process would be repeated for all samples in the dataset. Receiver-operator-characteristic (ROC) curves and the area-under-the-curve (AUC) were employed using the package pROC [10] in R to evaluate the classifier performance. AUC values were displayed in figures as mean ± confidence interval (95%).

### Calculation of interferon-stimulated genes’ scores

First, relative expression (RE) was calculated based on the readcounts of interferon-stimulated genes (ISG) and the housekeeping gene GAPDH: $\text{RE = }{\text{2}}^{\text{-}\left(\text{count ISG}-\text{count GAPDH}\right)}$. The ISG score was calculated by summing up the individual RE per gene after normalization to the control group as follows: $\sum \left({\text{RE}}_{\text{subject}} -\text{ }{\text{Mean}}_{\text{control}}\right)/{\text{Standard Deviation}}_{\text{control}}$. 28 ISG were selected for ISG score calculation (Table S4) [11]. Statistical significance was assessed using Mann-Whitney test. * *P* < 0.05; ** *P* < 0.005.

## Results

### Transcriptome profiles of rheumatic diseases, viral infection, and convalescent controls

**Fig. 1:**
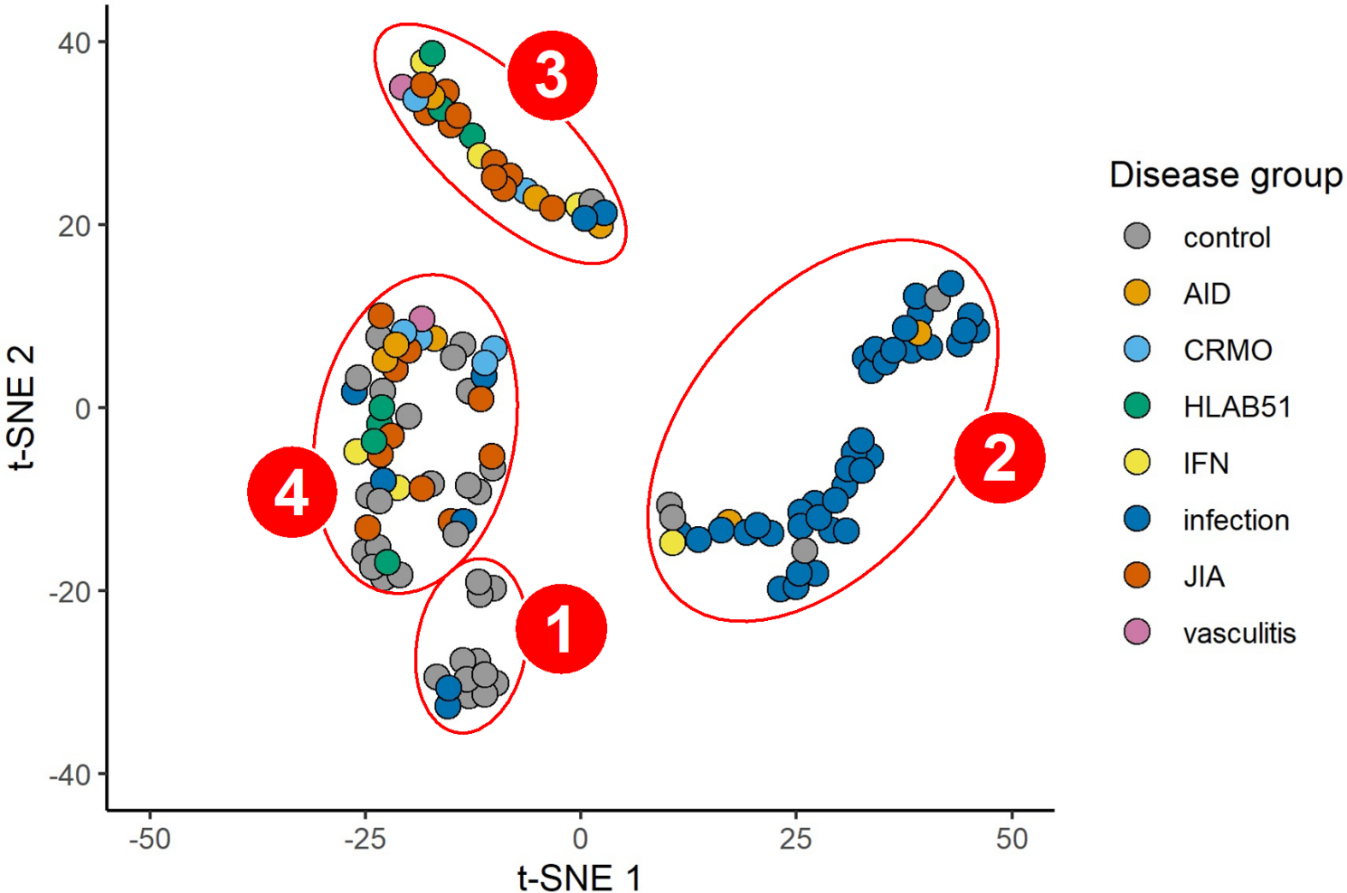
t-SNE plot of 4 different clusters

We compared the transcriptome profiles of six rheumatic disease groups (i.e., JIA, AID, CRMO, HLA-B51, IFN, and vasculitis) with viral infection and convalescent controls. Clustering analyses using t-SNE and hierarchical algorithms were displayed in Fig. 1 and Figure S1, respectively. As shown in the t-SNE plot in Fig. 1, most controls were gathered in cluster 1 while infection cases were grouped into a separate cluster 2, which implies that the gene expression of actively infected cases and remission cases (i.e., controls) is substantially independent despite coming from the same participants. However, patients with different rheumatic diseases were not well distinguished and assigned mostly to cluster 3, while cluster 4 contained a mixture of different categories.

### Classifier development

**Fig. 2:**
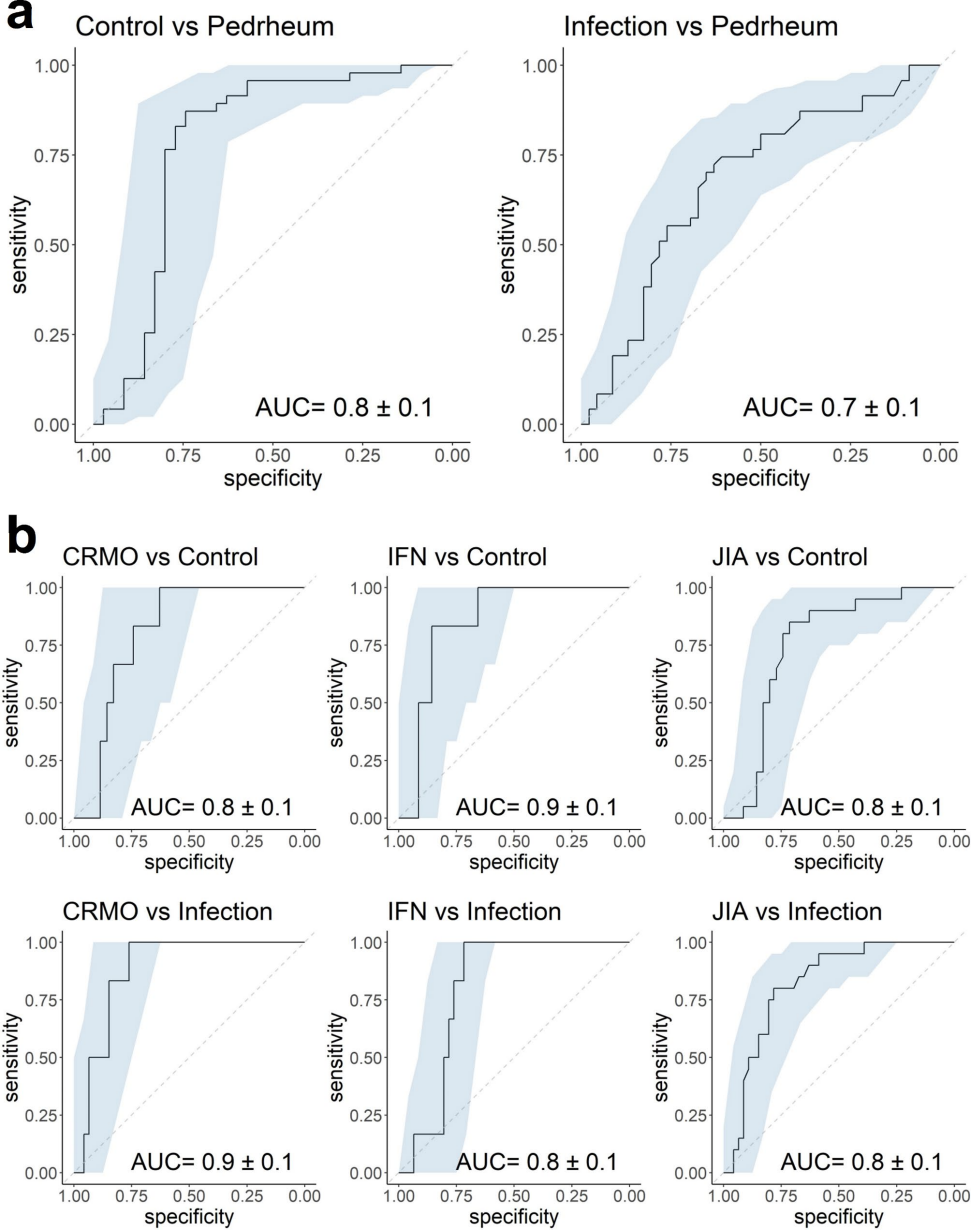
ROC curves and AUC values from leave-one-out cross-validation of classifier between (a) negative controls (i.e., control), viral infected subjects (i.e., infection) and subjects with rheumatic diseases (i.e., Pedrheum); and more specifically between (b) CRMO, IFN, JIA and control/infection cases

The Random Forest algorithm was used for classifier development because it uses the ensemble learning technique that is robust to outliers, stable with new data, and can handle non-linear correlations. The first classifier was developed to distinguish between control, infection, and pediatric rheumatic cases based on normalized transcriptome data. Leave-one-out cross-validation results in Fig. 2a confirm that the classifier could differentiate pediatric rheumatic patients from negative controls (AUC = 0.8 ± 0.1) and from viral infection cases (AUC = 0.7 ± 0.1). The Boruta algorithm selected 349 genes out of 31,319 initial genes (Table S2) for the training of this classifier between control, infection, and pediatric rheumatic cases. Some of the notable selected genes were CD3G, CD96, and CD200R1 (CD200 receptor 1). The gene CD3G encodes the CD3γ polypeptide, which forms a part of the CD3-TCR (T-cell receptor) complex. This complex plays an important role in antigen recognition and several intracellular signal-transduction pathways. This finding indicates that some of the rheumatic diseases are specifically connected to the alteration and malfunction of γ T-cells. Previous studies have also reported the association of γ and δ T-cells with (immunodeficiency and) autoimmune diseases [12]. CD96 is expressed on T-cells and natural killer cells. It belongs to a family of molecules that provide costimulatory and coinhibitory signals during T-cell activation. It was shown to inhibit the expansion and IL-9 production of Th17 cells and thus, reduce inflammation and pathogenicity [13]. CD200R1 is also expressed on T-cells, as well as myeloid cells. It was reported to alter the balance between Th17 cells and regulatory T-cells in SLE patients [14] and has also been confirmed as one of the genetic factors susceptible to JIA, especially oligoarticular JIA [15]. Aberrant expression of CD200R1 was shown to contribute to abnormal Th17 cell differentiation and chemotaxis in patients with rheumatoid arthritis [15].

More specific classifiers were then developed per disease group. As the number of rheumatic patients in our dataset was limited, these classifiers focused only on CRMO, IFN, and JIA groups, which had more subjects for model training and validation than the other disease groups. Three classifiers were developed to distinguish patients with CRMO (n = 6), IFN (n = 6), and JIA (n = 20) from control (n = 35) and infection (n = 46) cases. They worked quite well as their AUC values are above or equal to 0.8 (Fig. 2b). Since CRMO, IFN, and JIA were differentiated well from control and infection cases, it was subsequently important to examine how they could be distinguished from one another. ROC curves and AUC values of a classifier between CRMO, IFN, and JIA (Figure S2) indicated that IFN could be distinguished relatively well from CRMO and JIA (AUC = 0.7 ± 0.2/0.3), however CRMO is not easily differentiated from JIA (AUC = 0.5 ± 0.3), likely explained by the limited sample size. The Boruta-identified genes for these classifiers are also presented in Table S2. There were 349 selected genes from the CRMO-control-infection classifier, 247 genes from the IFN-control-infection classifier, and 286 genes from the JIA-control-infection classifier. As expected, more interferon-related genes were selected for the IFN classifier compared to those of CRMO and JIA.

### Differential expression and gene ontology enrichment analyses

**Fig. 3:**
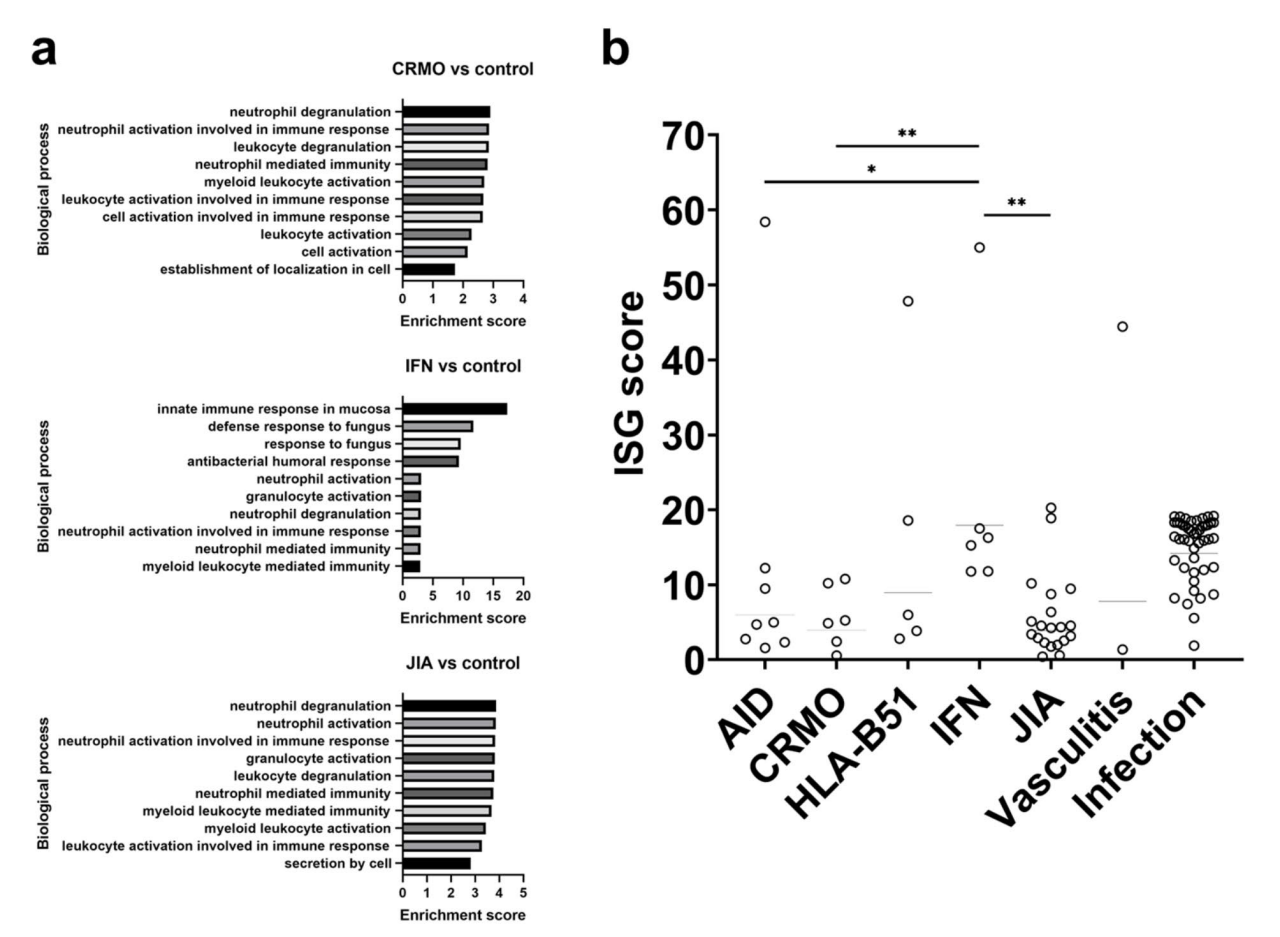
(a) Gene ontology enrichment analysis of CRMO-, IFN-, and JIA-associated genes. Bar charts the top 10 GO terms for biological process. (b) ISG score by disease group; horizontal lines represent median values of each group; Mann-Whitney test for statistical significance: * P < 0.05; ** P < 0.005

We analyzed the differentially expressed genes (DEGs) of CRMO, IFN, and JIA versus controls. The resulting DEGs were translated to corresponding Gene Ontology (GO) categories to understand which pathways were involved in the disease pathophysiology. Many of the top 10 GO categories of CRMO, IFN, and JIA groups are related to innate immunity including myeloid leukocyte and granulocyte activation, neutrophil activation and degranulation (Fig. 3a and Table S3). In IFN particularly, the immunity is largely mediated by antibacterial and antifungal defense responses. Results from GO analyses of CRMO, IFN, and JIA against the other Pedrheum groups are displayed in Figure S3 and Table S3. Although the classifiers could not adequately differentiate between CRMO and JIA, we noted that 1,106 DEGs could be found between CRMO and all other Pedrheum groups, 1,730 DEGs in the case of IFN, and 1,216 DEGs for JIA (Table S5). Additionally, more than 170 DEGs were found between CRMO and IFN, CRMO and JIA, as well as between IFN and JIA (Table S5).

### ISG scores

Using the whole blood gene expression obtained from 3’ mRNA sequencing, we calculated the ISG scores of IFN patients and compared them with those from other disease groups. As displayed in Fig. 3b, IFN patients had the highest mean score of 18. Other disease groups, although displaying lower mean scores than IFN (6.0 for AID, 4.0 for CRMO, 9.0 for HLA-B51, 3.9 for JIA, 7.8 for vasculitis, and 14 for infection cases), did include some patients with particularly high scores: one AID patient had a score of 58, one HLA-B51 patient had score 48, and one vasculitis patient had score 44. Interestingly, longitudinal tracking of ISG scores was proven feasible using 3’ mRNA sequencing. Indeed, we showed that one patient with Aicardi-Goutières syndrome had significantly high ISG scores at early presentations that decreased following initiation of JAK-inhibition via tofacitinib (see Figure S4).

## Discussion

Application of transcriptomics techniques such as microarray or sequencing on blood or synovial fluid of rheumatic patients has been a key transforming factor in rheumatology [1]. In the current study, we showed that 3’ mRNA from whole blood can provide adequate information for the differentiation between controls, viral infections, and pediatric rheumatic diseases. We demonstrated that the Random Forest algorithm can be applied on blood transcriptome data to determine whether a pediatric patient has reactive/post-infection phenomena or an autoimmune/autoinflammatory disease. Furthermore, studies on autoimmune and autoinflammatory diseases have largely focused on adaptive immunity, that is, regulatory and autoreactive T-cells [16]. Our study hereby provides evidence that innate immunity may also have an important role in the pathophysiology of pediatric rheumatic autoimmune and autoinflammatory diseases, including CRMO, IFN, and JIA. Moreover, we showed that the activation and immune response of myeloid cells form participate in the biological pathways underlying JIA, CRMO and IFN – three of the most common rheumatic diseases in children. Via the DEG and GO analyses, we found that the immunological activities of innate cells, such as neutrophils and granulocytes, were highly associated with CRMO, IFN, and JIA compared to the viral convalescent controls. Interestingly, in the IFN group, the immunity seems to be largely mediated by antibacterial and antifungal defense responses. This could be a sign of molecular mimicry where self-derived peptides resemble foreign antigens and thus stimulate the antigen-specific autoreactive T-cells or B-cells, which in turn results in the production of pro-inflammatory cytokines [17, 18]. One of them is type I interferon, which plays an important role in the pathophysiology of IFN disease and which also has been reported to participate in the immune response against viral, bacterial, fungal pathogens, and parasites [19]. GO analyses comparing CRMO, IFN, and JIA with the other Pedrheum groups (Figure S3 and Table S3) reveal that CRMO is specifically driven by cellular response to cytokine, JIA by cellular response to chemical stimulus, and IFN by the activation of myeloid leukocytes, neutrophils, and granulocytes. The dysregulated cytokine expression from innate immune cells has been concluded to have central contribution to the inflammatory phenotype of CRMO by Hofmann et al. [20]. JIA on the other hand has been found to be associated with antibiotics exposure in a dose- and time- dependent fashion in a large pediatric population by Horton et al. [21].

The over-expression of ISG is a useful biomarker of IFN diseases, including SLE. Although the interferon signature was first defined in SLE patients, different ISG are investigated to classify pathological conditions of other interferonopathies and lupus-like disorders (e.g., dermatomyositis, Aicardi-Goutières syndrome) to guide molecular diagnostics and to formulate targeted therapy approaches [22]. Quantitative polymerase chain reaction (qPCR) has been the method of choice to measure the expression of ISG and estimate the ISG score, but this approach has low throughput as qPCR can only analyze pre-determined genes and the number of genes to be analyzed simultaneously is limited [23]. Using whole blood gene expression obtained from 3’ mRNA sequencing, we were able to calculate the ISG scores without gene pre-selection or gene number limitation. Our data show that the average score of IFN group was the highest among all disease categories, thereby confirming that the highly expressed ISG are signatures of interferonopathies. More importantly, we showed that robust longitudinal tracking of ISG scores is possible with the use of 3’ mRNA sequencing. Beside the IFN group, viral infection cases also displayed high average ISG scores due to the participation of type I interferon in the immune responses against viral infection [19].

Despite demonstrating that blood RNA sequencing can distinguish autoimmune/autoinflammatory diseases from viral infection/post-infection cases, as well as reveal their key genes and pathways, our study is limited by the sample size of each rheumatic disease, due to which cross-validation was done to validate the classifiers’ performance instead of training and testing on independent datasets. The limited number of rheumatic cases may also be responsible for the modest cross-validation performance of the CRMO-IFN-JIA classifier although rheumatic diseases in general could be identified well from controls and infections. Another limitation that challenges the predictive performance of our classifiers is disease heterogeneity. IFN and JIA are examples of heterogeneous groups that pose great difficulties for the classifiers to differentiate from other groups because they contain several disease subgroups (e.g., dermatomyositis and systemic lupus erythematosus were grouped together as IFN, and JIA included polyarticular, oligoarticular types, as well as spondyloarthropathies). The heterogeneity of diseases often obstructs explicit modelling of underlying distributions of individual features, which can be even more problematic when the sample population is small [24]. Finally, although efforts were made to minimize batch effects, these cannot always be completely avoided.

To incorporate the expression-based assessment into clinical use, it is necessary to demonstrate that the reliability and accuracy of this approach is comparable or superior to the currently used methods. The development of diagnostic criteria is challenging in rheumatology due to the heterogeneity of many rheumatic diseases, variable clinical presentations, and complex pathophysiology. Given the lack of optimal diagnostic criteria, physicians must rely on a complicated decision-making process based on a combination of symptoms, physical examination, exclusion of competing diagnoses, and geographic prevalence [25]. Moreover, there is an ongoing concern that physical examination is insensitive in detecting subtle, smoldering synovitis [26]. Since the completion of the Human Genome Project in 2003, next-generation sequencing has seen great improvements in technique and decline in cost. In addition, the whole protocol from RNA extraction, library preparation, and sequencing until data pre-processing and disease classification using machine learning only takes 4–5 days in our experience, given that a classifier is already developed and validated. This is a similar average amount of time it would take to complete a blood test and other assessments for the diagnosis of rheumatic diseases. Thus, the approach proposed in this study, where machine learning is developed based on blood transcriptome data, should be highly affordable and applicable in clinical practice. Since gene expression variations in blood cells of rheumatic patients can predate the clinical manifestations [27], blood gene expression profiling is useful in identifying new biomarkers of pediatric rheumatic diseases and, together with the machine learning classifiers presented in this study – after further development and validation – will be an efficient tool for early diagnosis and heterogeneity exploration of pediatric rheumatic diseases. We believe that adding more cases of pediatric rheumatic diseases to the database will provide more data for the classifier to be trained on, allowing it to capture more distinct transcriptome features and variances of each disease, as well as get validated on an independent test set. It would also be useful to collect blood from the same patients over fixed time periods or before and after therapy to obtain longitudinal transcriptome data so that we can update and improve the model to foresee patients’ clinical course or treatment response.

## Conclusion

Overall, our study indicates that blood transcriptomics is a promising tool to improve the diagnosis of pediatric rheumatic diseases. The ease of sample collection as well as the continuous enhancement and affordability of sequencing techniques can overcome the challenges of patient heterogeneity and allow for further fruitful research.

## Electronic supplementary material

Below is the link to the electronic supplementary material.

Supplementary Material 1

Supplementary Material 2

Supplementary Material 3

Supplementary Material 4

Supplementary Material 5

Supplementary Material 6

## Data Availability

The datasets used and/or analyzed during the current study are available from the corresponding author upon reasonable request.

## Abbreviations

AID: Auto-Inflammatory Diseases
AUC: Area Under the Curve
CRMO: Chronic Recurrent Multifocal Osteomyelitis
DEG: Differentially Expressed Gene
GO: Gene Ontology
HLA-B51: Diseases related to the Human Leukocyte Antigen B51 serotype
IFN: Interferonopathy
IL: Interleukin
ISG: Interferon-Stimulated Gene
JIA: Juvenile Idiopathic Arthritis
RE: Relative Expression
RNA: Ribonucleic Acid
ROC: Receiver-Operator-Characteristic curve
SLE: Systemic Lupus Erythematosus
t-SNE: t-distributed Stochastic Neighbor Embedding method
