# Supplementary material for: Blood transcriptomics to facilitate diagnosis and stratification in pediatric rheumatic diseases – a proof of concept study
Source: Pediatr Rheumatol Online J. 2022 Oct 17;20:91. doi: 10.1186/s12969-022-00747-x (PMC9575227; doi:10.1186/s12969-022-00747-x)
Supplement: Supplementary file 1 — Supplementary Material 1 [file 12969_2022_747_MOESM1_ESM.docx]

**Blood Transcriptomics to Facilitate Diagnosis and Stratification in Pediatric Rheumatic Diseases**

My Kieu Ha^1,2,3,*^; Esther Bartholomeus^2,3,4^; Luc Van Os^5^; Julie Dandelooy^6^; Julie Leysen^6,7^; Olivier Aerts^6,7^; Vasiliki Siozopoulou^8^; Eline De Smet^9^; Jan Gielen^9,10^; Khadija Guerti^11^; Michel De Maeseneer^12^; Nele Herregods^13^; Bouchra Lechkar^14^; Ruth Wittoek^15,16^; Elke Geens^16^; Laura Claes^17^; Mahmoud Zaqout^18,19^; Wendy Dewals^18^; Annelies Lemay^20^; David Tuerlinckx^21^; David Weynants^22^; Koen Vanlede^23^; Gerlant van Berlaer^24^; Marc Raes^25^; Helene Verhelst^26^; Tine Boiy^27^; Pierre Van Damme^2,28^; Anna C. Jansen^17^; Marije Meuwissen^3^; Vito Sabato^14,29^; Guy Van Camp^4^; Arvid Suls^2,4^; Jutte Van der Werff ten Bosch^30^; Joke Dehoorne^31^; Rik Joos^16,27,29,31^; Kris Laukens^2,32,33^; Pieter Meysman^2,32,33^, Benson Ogunjimi^1,2,16,27,29,34^

* Corresponding author: My Kieu Ha

Faculty of Medicine and Health Sciences

Vaccine & Infectious Disease Institute

Campus Drie Eiken – building S, room 2.42

Universiteitsplein 1, 2610 Antwerp (Wilrijk)

Email [my.ha@uantwerpen.be](mailto:my.ha@uantwerpen.be)

**
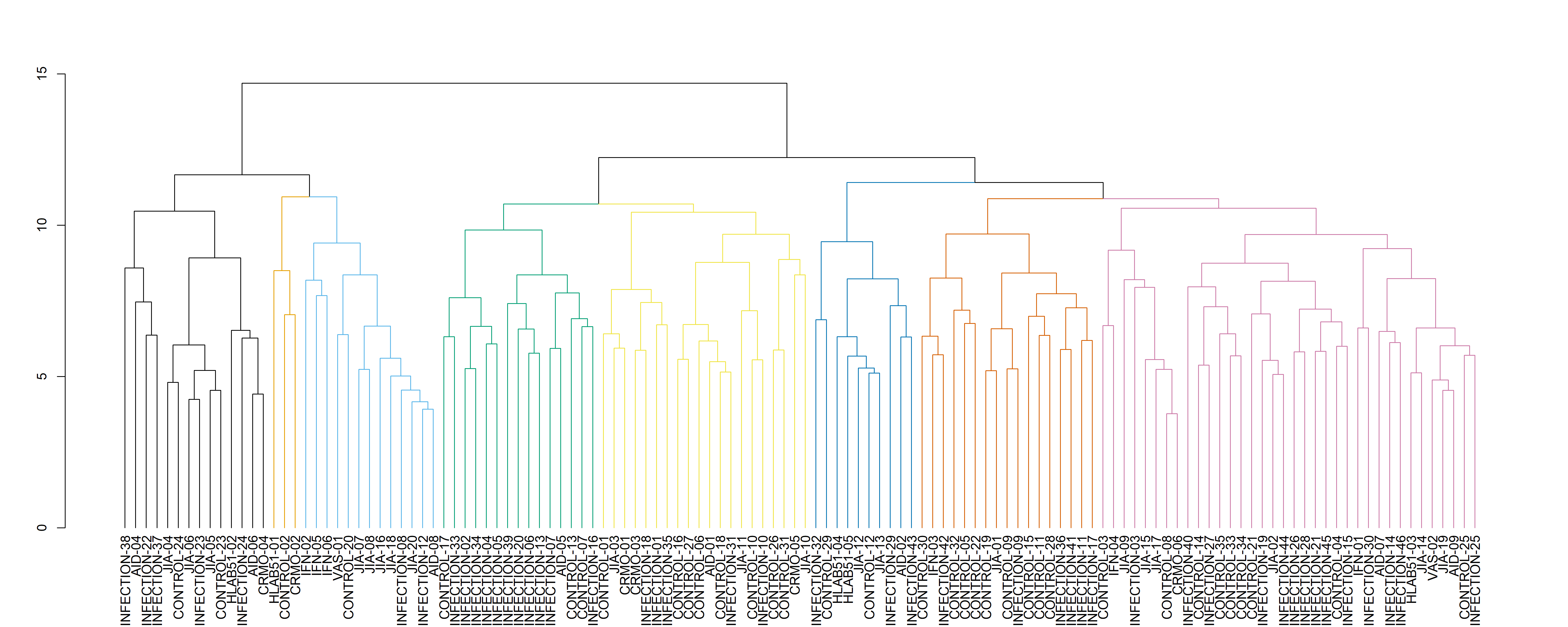
**

Supplementary Figure S1. Hierarchical clustering groups different participants into 8 clusters, identified by their colours..

**
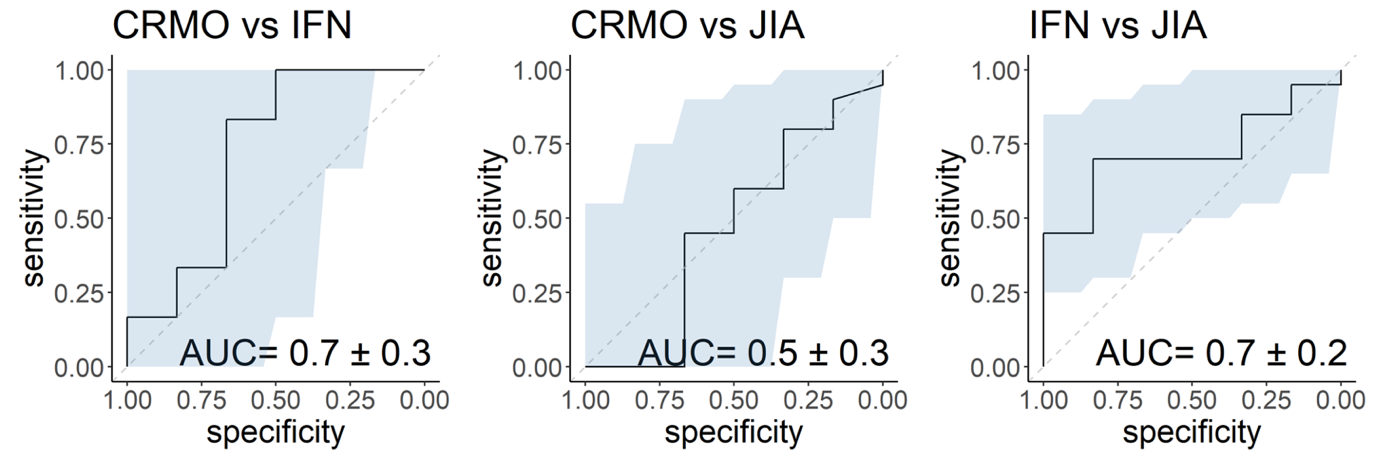
**

Supplementary Figure S2. ROC curves and AUC values from leave-one-out cross-validation of classifiers between CRMO, IFN, and JIA.


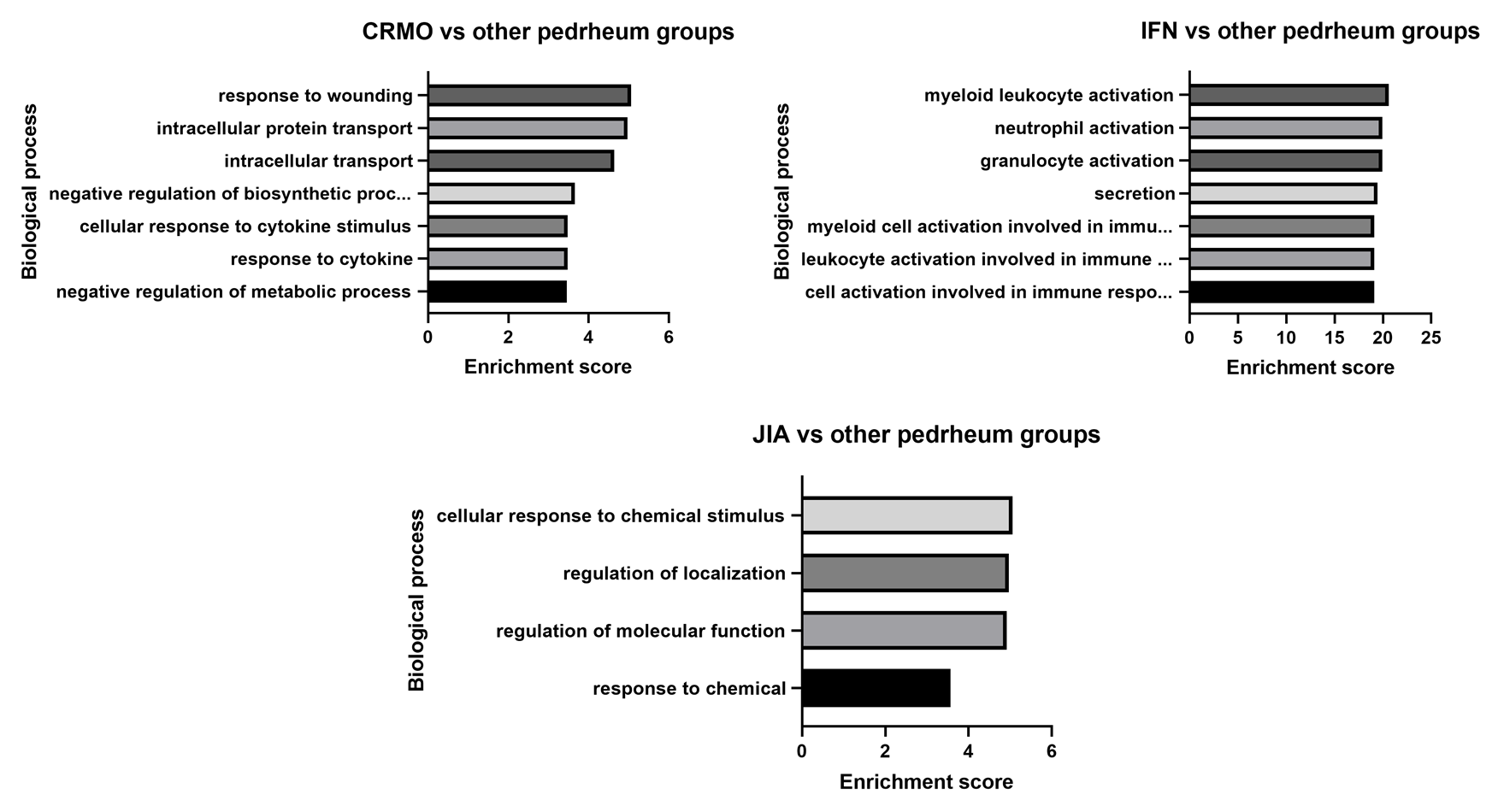


Supplementary Figure S3. Gene ontology enrichment analysis of CRMO-, IFN-, and JIA-associated genes. Bar charts the top GO terms for biological process. Complete lists of significant GO terms are in Table S3.


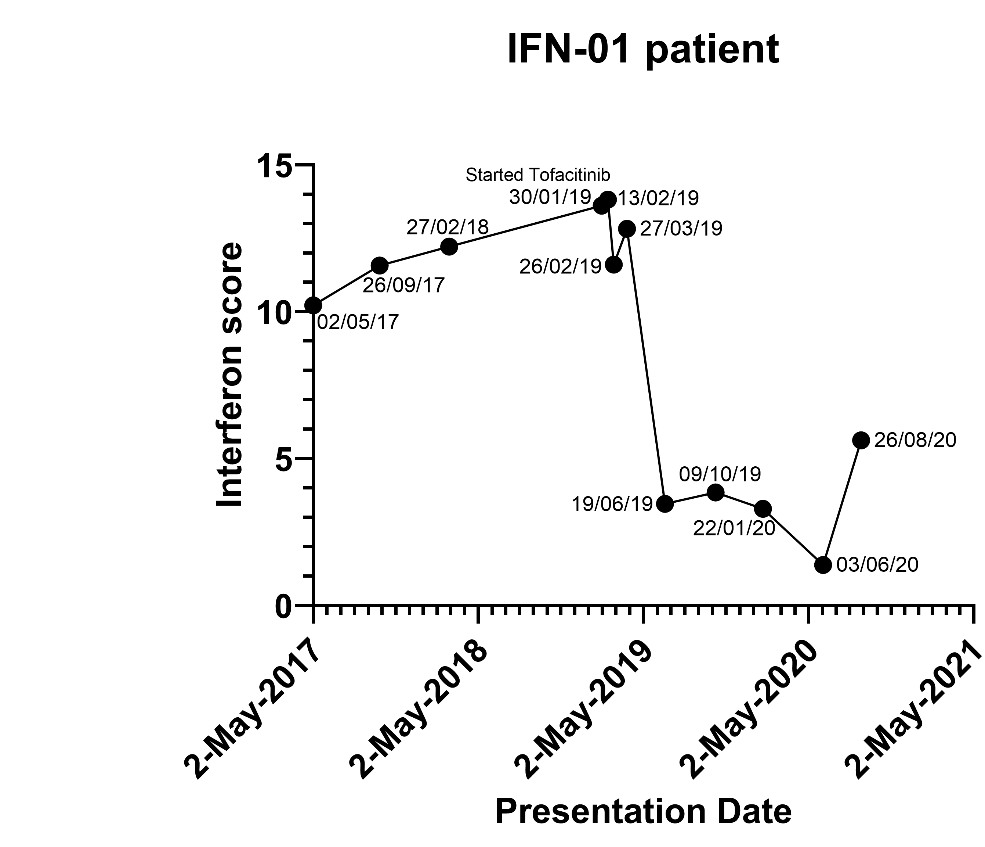


Supplementary Figure S4. Longitudinal interferon scores of IFN-01 patient throughout the 3-year treatment. Tofacitinib medication started on 13-Feb-2019. The patient received hydroxychloroquine between 27/2/2018 and 7/12/2018.
